# Poly-Pharmacy

**Published:** 1873-12

**Authors:** Henry B. Upton

**Affiliations:** Osceola, Ill.


*MEDICAL EXAMINER—December*, 1873:

Poly-pharmacy—*By Henry B. Upton, M.D., Osceola, Ill.*—The effort to advance medical science by experimental practice brings to our notice some new remedies every year; and yet disease and death, unmindful of their claimed potent influence, march on unchecked. Could we, as a profession, supply with our powders, pills, solutions, and decoctions, nerve-force as readily as the engineer gives power to his machine, we would be most fortunate indeed.

I am frequently inclined to think that we have *too much materia medica* and *too little common sense.* I am fully convinced that many of the substances held in high repute by medical professors, and upon which we have often relied as possessing wonderful remedial power, are almost, if not totally, inert, so far as any action upon the functions of life are concerned. I have treated cases where ordinary doses of opiates were powerless, but bread pills and small doses of aqua pura, administered with punctilious regularity, were all-powerful and magical in their action. While I would not condemn at one sweep the therapeutics of our accepted materia medica, I would most earnestly caution against the too implicit trust sometimes reposed in their power. The most powerful medicines are the most poisonous, and should be most carefully applied. Meddlesome application of materia medica is as bad as meddlesome midwifery ; and the way some of the victims of the profession have been stuffed with villainous compounds, and abluted with multitudinous mixtures—oleaginous, alcoholic, alkaline and aquæous (frequently the quadruple combination)—has been sufficient to carry many to their last long home, where, if they wake to consciousness, they will hope no doctors may come. Still, many recover in spite of wholesale medication—spared monuments, not of the doctor's skill, but of the great recuperative power of nature. The very fact that different members of this Association treat the same abnormal conditions of the human body with such a diversity of remedies, often absolutely opposite in their action, utters volumes in denunciation of any course of practice trusting implicitly upon the virtue in our materia medica. It is more than possible that those who recover would do so without the aid of a physician, although it might not be in a strictly scientific manner.

